# Supplementary material for: Universities’ Scientific and Technological Transformation in China: Its Efficiency and Influencing Factors in the Yangtze River Economic Belt
Source: PLoS One. 2021 Dec 16;16(12):e0261343. doi: 10.1371/journal.pone.0261343 (PMC8675682; doi:10.1371/journal.pone.0261343)
Supplement: Attachment — Submitted filename: Response to Reviewers 2021-6-23.docx [file pone.0261343.s002.docx]

**Dear the Editor,**

**Thank you very much for the comments of the reviewer. We have carefully revised the manuscript. Our responses are as follows:**

- Question1: In the abstract, the authors write "Avoiding the problem of considering the input and output of university S&T transformation as a "black box" and neglecting the links among different transformation stages.". However, it is well known that DEA methods (including SBM) have the disadvantage of considering the production process as a black box. I would like the authors to explain this conclusion.

**Reply: Thanks for the suggestions. Based on the suggestions from the review, we carefully rewrite the method part of the abstract, and consistently reorganize the method part, in order to avoid the uncertainty of method description. The revised part as follow:**

*Abstract: Universities are important sources of knowledge and key members of the regional innovation system. The key problem in Chinese universities is the low efficiency of the scientific and technological (S&T) transformation, which limits the promotion of regional innovation and economic development. This article proposes the three-stage efficiency analytical framework, which regards it as a complex and interactive process and obtain more objective and accurate efficiency measurement. This paper applied super efficiency network SBM model which contains input and output slack variables, so the model can be used to measure efficiency more accurately. Empirical research proves that university S&T transformation has not been effectively improved and the scientific resources invested in universities have not been efficiently utilized in recent years. Generally, Despite the correlation between regional economy and transformation efficiency, the exclusive increase in resources is not enough. Regional openness and the quality of research talents are key factors for the application of technological innovation and technology marketization. Universities should not only pursue the number of research outputs but pay more attention to high-quality knowledge production to overcome difficulties in research achievements transformation.*

*Method: DEA (Data Envelopment Analysis) [20-21] and SFA(Stochastic Frontier Analysis) [22-24] have been used to measure the efficiency of S&T achievements transformation in western developed countries and determined the factors restricting the transformation process. DEA is a scientific method for efficiency evaluation, which avoids the inaccuracy of a subjective weight setting. However, in empirical research, the input and output of DEA mainly focused on the initial input resources and final output[25]. The traditional radial DEA model will lead to efficiency measurement deviation due to the difference of radial selection in efficiency evaluation. The objective function of SBM model contains input and output slack variables, which can be used to measure efficiency more accurately. With the optimization of efficiency measurement model, based on the innovation process analysis framework, the efficiency research is divided into different stages for evaluation [26]. Focus on the sub stages of innovation process, and establish the relationship among different stages [27]. According to the transformation chain of university S&T achievements, the input-output division is divided into three stages; this method allows us to systematically grasp the total efficiency of the transformation chain and accurately analyze the efficiency problems in different transformation stages.*

- Question2: Please describe in detail the embedding position of this paper in the existing studies and the marginal contribution made by this paper.

**Reply: Thank you for the good advice, which help a lot for deep thinking for both the theory and practical meaning and contribution of the research. We add the main contribution of this paper at the conclusion and discussion part. It helps to understand the main theory and practical significance Following are the added parts:**

*Interrelationship of science and technology between university and industry has been admitted over time [42]. It is used to explain organizational relationship between public research and industry, especially how they interact to realize knowledge transfer and innovation [43]. Scientific and technology knowledge are interacted over time, and university are important actor in knowledge transfer system, which can cooperate with firms and influence regional development. Therefore, the analysis and mechanism exploration of university scientific creation, technology application and marketization progress will help to understand and improve regional innovation.*

*Overall, this paper mainly contributes to the existing research on university S&T performance in the following points. Firstly, this research focus on a representative heterogeneous region in china, wish to help improve the overall performance of* *university S&T not only in the economic core region, but also some relatively peripheral regions. Secondly, this research not only analyze influence factor of the overall transform performance, but performance of basic knowledge acquisition, applied technology innovation and technology marketization stages, mainly to accurately reflects the main problems in different process of university S&T transformation. The third point is that by apply super-SBM model, we made more objective and distinctive values of university S&T transformation. Totally, this research has significance for empirical analysis on university S&T transformation and marketization in China's heterogeneous regions.*

- Question3: The introduction of the Yangtze River Economic Belt in section 4.1 is better moved to the background section.
- Question4: What are the standards for dividing the central and western regions in the paper? Why are there only 3 and 4 provinces in the central and western regions respectively? In addition, the result that efficiency is higher in the western region than that in the central region is inconsistent with experience, please provide an explanation.

**Reply: Thanks for the suggestions. We firstly reorganized the order of the paper, move section 4.1 to the introduction part which is the background part of this paper.**

**Secondly, we add more specific explanation about how and why we divided the region into three parts. The selection and division criteria of regions are mainly based on the national planning and related research. Because we focus on the Yangtze River Economic Belt (including 11 provinces and cities), not the whole of China. Therefore, there are only 3-4 provinces in each of the three major divisions within the economic belt. The revised and added parts are as follow:**

*The promotion of the development of the Yangtze River Economic Belt is a national strategy that affects the development of China. Yangtze River Economic Belt includes 11 provinces and cities: Shanghai, Jiangsu, Zhejiang, Anhui, Jiangxi, Hubei, Hunan, Chongqing, Sichuan, Yunnan, and Guizhou. According to the regional planning policy and existing literature [34,], this paper divide the Yangtze River Economic Belt mainly into three heterogeneous region. Region1 (according to “Yangtze River Delta Regional Planning (2010)”), there are one direct-controlled municipality and*

*two provinces, Shanghai, Jiangsu, and Zhejiang; Region 2 (Region 2 contains four provinces: Anhui, Jiangxi, Hubei, and Hunan) is located in midland China, acts as a bridge between east and west China; Region 3 (Region 3, western metropolis area, contains three provinces and one direct-controlled municipality: Chongqing, Sichuan, Yunnan, and Guizhou) is economic periphery region in the west of China. The Yangtze River Economic Belt accounts for 21% and 43% of China’s land area and population, and it is a representative region of innovation in China. Comparing the R&D proportion of the Yangtze River Economic Belt with that of the entire country, the proportion of R&D expenditure in the Yangtze River Economic Belt accounts for 46% of China’s expenditure, and the R&D personnel accounts for 45%.*

*The spatial correlation analysis of the innovation output shows that Moran’s I index of the Yangtze River Economic Belt is all positive, and spatial dependence characteristics of the innovation output are becoming obvious. According to Lisa (Local indicators of spatial association) analysis, the high-value output agglomeration of the Yangtze River Economic Belt is not significant; Shanghai, Suzhou, Ningbo, Hangzhou, Wuhan, Changsha, and Chongqing show higher value characteristics than surrounding cities. The high-value cluster of the innovative output continues to expand northward in Region 1, whereas the level of innovation output between Region 1 and Region 2 tends to be similar, but the level of agglomeration is relatively low (Figure 1).*

*Given the huge regional heterogeneity, differences in innovation volume among regions are inevitable. Therefore, when analyzing areas with large spans and significant internal differences, we should not only focus on the absolute amount of resources but also consider whether knowledge or resources are reasonably configured. Therefore, when analyzing the S&T efficiency of universities and the influencing factors, this paper considers regional heterogeneity, analyzing the overall efficiency and evolution characteristics of the heterogeneous region.*

**Thirdly, we add explanation about why the western region is a bit higher than that of the middle region. The explanation based both on the result and the existing research about the belt region, in order to prove that this result is actually consisted with the demand and characteristics of regional innovation. The added parts are as follow:**

*The overall efficiency of the western region is higher than that of the central regions, and the universities in Yunnan and Guizhou have higher efficiency. Through the research of existing literature on the path of regional technology transformation, it is pointed out that the role of public research institutions such as universities in the western region is more significant than that in the central region [41]. The purpose of basic knowledge cooperation in the western region can directly affect the development of regional industrial economy, and can also promote the regional industrial economy through the application-oriented transformation or market-oriented value of basic knowledge. Moreover, compared with the central region, the western region's knowledge application and technology transformation are more realized by colleges and universities, which also explains why the efficiency of knowledge transformation of colleges and universities in the western region is higher than that in the central region. This is consistent with the conclusion that the main goal of inter-regional network innovation cooperation in western regions is to realize the new technology application and marketization (Lin, 2016).*

- Question5: Please elaborate on the criteria for the selection of explanatory variables in panel regressions.

**Reply: Thank you for the advice. In order to help understand why are these indicators selected. We add explanation and also a new table to the data and method part. The added parts are as follow:**

*The influencing factors of the university S&T achievements transformation involve complex aspects.It has been pointed out that the process of technology transformation includes complex external environment, and the influencing factors include many aspects. Most of the existing researches take regional macroeconomic environment, external environment, regional science and technology environment as different indicators [1-3]. The macro-economic environment can reflect the local market demand scale, industrial structure and payment ability of universities in the process of scientific and technological transformation; External environment can reflect regional foreign exchange ability and openness, which will affect the exchange and promotion of regional scientific and technological achievements; Regional science and technology environment directly affects the flow of talents and the support for the scientific and technological transformation. Therefore, based on the existing research on the selection of influencing indicators, this paper selects specific indicators from the above three aspect (Table 2).*

*Based on existing research and considering the reality of Chinese universities and data availability, the transformation efficiencies of three stages are selected as dependent variables. including the regional economic foundation, openness, industrial structure, quality of scientific research personnel, scientific research projects, and university international cooperation as the influencing factors. The panel regression model of the influencing factors on the university S&T achievements transformation progress is as follows:*

**Table.2 Indicator of influence factor of Transform Efficiency**

| **Factor** | **Index** |
| --- | --- |
| Macroeconomic  Environment | regional gross domestic product;  regional industry structure; |
| External  Environment | foreign direct investment;  university cooperation; |
| Technology  Environment | quality of R&D talents;  university scientific research project; |

*(1) GDPit: The regional economic level shows a certain correlation with S&T transfer. The GDP is taken as the measurement index to test the influence of regional economic development on the transformation efficiency of university S&T.*

*(2) INDit: There may exist a certain correlation between industrial structure and the transformation of S&T. Based on existing research, this paper uses the proportion of tertiary industry to measure the optimization degree of industrial structure.*

*(3) OPENit: Regional universities usually guarantee their competitiveness in the region by local resources investment. After entering the international market, universities must also actively transform technological achievements and carry out more efficient innovation activities to meet the demands of international competition. In this paper, FDI (Foreign Direct Investment) is used as an important indicator of regional international openness.*

*(4) INTit: International cooperation between universities is important to developing countries to absorb new knowledge and technology. The number of international exchanges and cooperation in universities is used to measure university international cooperation.*

*(5) RESit: The number of scientific projects could reflect the scientific research ability of universities. Therefore, this number is selected as an index to measure the university S&T transformation efficiency.*

*(6) TALit: The quality of R&D talents is closely related to S&T transformation. In this paper, the proportion of R&D personnel with senior titles is used to measure the quality of R&D talents.*

- Question6: Why do almost all factors have no significant effect on the efficiency of the third phase?
- Question7: Regression results after adding fixed effects should be reported in table 3.

**Reply: Thank you for the questions, which help us rethinking our research deeper. On the one hand, we reorganized and summarize the explanation of the influence factor, based on existing research about innovation and tech transformation and combined with regional features. On the other hand, as the fix or random effect in panel regression is based on value of hausman test, so we show the result value of hausman test in order to prove the fix model of the first and second stage. We also add explain about the fix effects to make it clear for the reader to understand. Revised and added parts are as follow:**

*In this part, we applied panel regression model to find out the main influencing factors of university S&T transformation. Based on result of hausman test, transformation of basic knowledge acquisition stage and technology application stage are fitted for fixed efficiency, the marketization stage is fitted for random efficiency (Table 3).*

*According to the panel regression analysis, regional GDP and foreign direct investment are main factors that influence basic knowledge acquisition stage; FDI and quality of talents have strong positive influence on technology application stage; Moreover, the quality of talents also influence technology marketization stage. From the perspective of technology marketization in universities, it is consistent with the relevant research conclusions of technological innovation and transformation in the Yangtze River economic belt[34,41]. Most universities need to cooperate with enterprises in technology transformation, and inter-regional cooperation is an important way. Especially in the central and western regions of the Yangtze River, universities realize technology marketization by cooperation with technology enterprises in the Yangtze River Delta. Therefore, for the marketization of technological achievements of colleges and universities in the Yangtze River economic belt, the regional macroeconomic environment and international investment do not have a direct and significant impact on the transformation efficiency. The talents of the region itself and the cross regional relations that these people can establish will have a great impact on the central and western regions.*

**We are thankful for the review and editor，the suggestions for this manuscript help a lot to make the article step further.**

**Best regards!**

**All authors of the article**

**2021-6-23**
